# Long‐standing hypercalcemia in a 78‐years old woman

**DOI:** 10.1002/ccr3.3994

**Published:** 2021-02-24

**Authors:** Andreas Kiriakopoulos, Dimitrios Linos

**Affiliations:** ^1^ 5^th^ Surgical Clinic Department of Surgery “Evgenidion Hospital” National and Kapodistrian University of Athens Medical School Athens Greece; ^2^ Department of Surgery “Hygeia Hospital” Athens Greece

**Keywords:** neck exploration, neck U/S, parathyroid adenoma, parathyroidectomy, primary hyperparathyroidism, sesta‐MIBI scanning

## Abstract

A 78 years‐old woman was found with worsening hypercalcemia, osteopenia and memory loss during the past 2 years. Multiple, repeated imaging studies failed to reveal the etiology of the primary hyperparathyroidism. Bilateral neck exploration revealed a 4.5 × 2.3 cm right superior parathyroid adenoma in an ectopic position.

## Graphical abstract

Primary hyperparathyroidism is the most common cause of hypercalcemia. Negative imaging studies neither exclude the diagnosis nor contraindicate the operative approach. Bilateral 4‐gland exploration offers the best chance of cure in this setting.

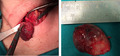

## 1 CASE PRESENTATION

**FIGURE 1:**
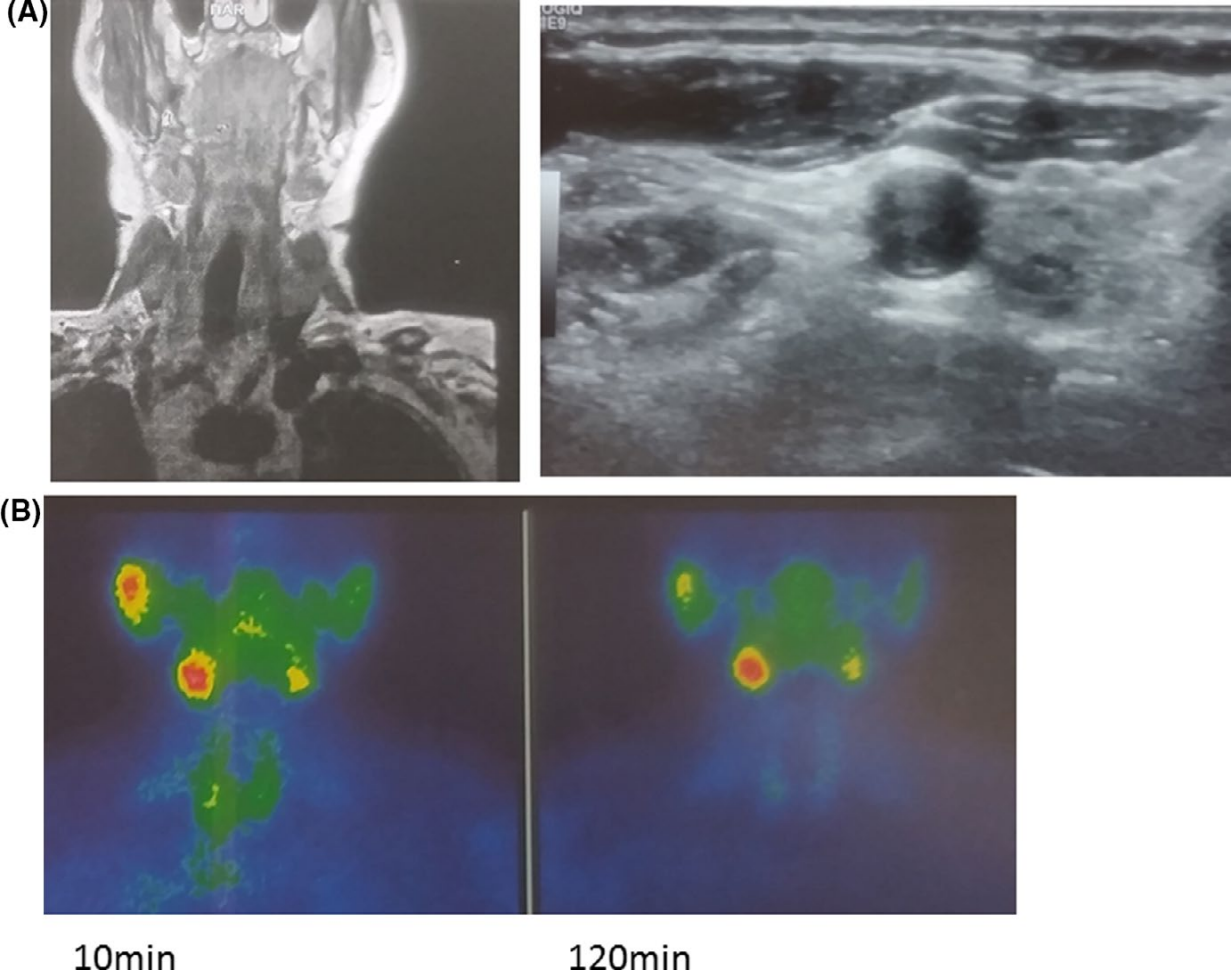
A, Neck CT scanning with IV contrast and transverse neck U/S showing no pathologic findings from the parathyroid glands. B, Sesta‐MIBI scanning 10 min and 120 min after the injection of the radiotracer without pathologic focal uptake

A 78‐year‐old woman was referred due to worsening hypercalcemia during the past 2 years. She complained of memory loss and was found with osteopenia (hip T‐score: −2.3 on DXA scan). Her past medical and family history was unremarkable. Calcium and PTH serum levels were 11.6 mg/dL and 156 pg/mL 2 years prior and 13.6 mg/dL and 662 pg/mL on admission (normal values: 8.4‐10.1 and 15‐65, respectively). Albumin, vitamin D, and kidney function were normal. Neck ultrasound/CT/MRI and sesta‐MIBI did not found any pathologic findings (Figure 1A,B).

### 1.1 Q: What is the diagnosis?

**FIGURE 2:**
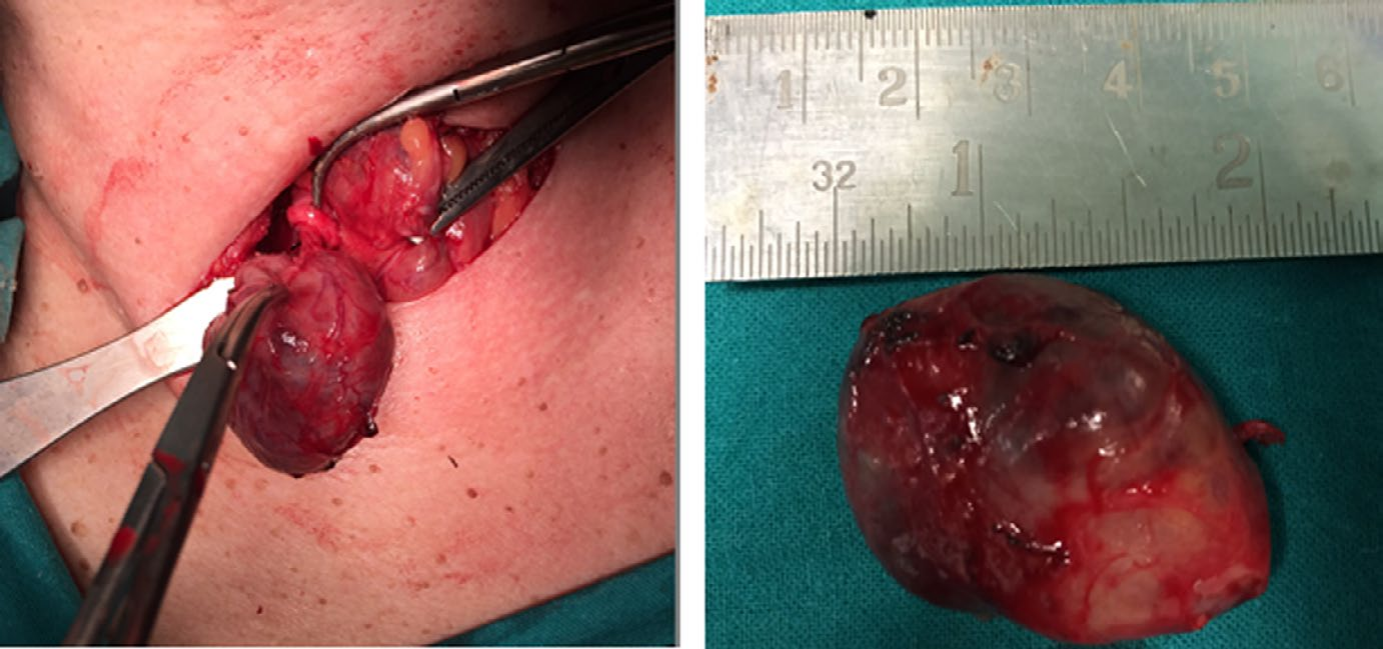
Large right superior parathyroid adenoma found at the lower right paratracheal area

Bilateral 4‐gland exploration revealed a right superior parathyroid adenoma at the lower paratracheal area (Figure 2). Histology confirmed the presence of an encapsulated, 10 gr/4.5 × 2.3 cm, parathyroid adenoma. A peripheral rim of normal glandular tissue was identified.

Primary hyperparathyroidism is the most common cause of hypercalcemia in the adult population. The diagnosis is biochemical and entails an inappropriately high PTH level despite high‐normal/high Ca^++^ serum level. Secondary causes of PTH elevation should be ruled out. Imaging studies do not aid in the diagnosis and are not used for selecting patients for surgical referral.^1^ Even though various minimally invasive techniques do exist, open bilateral 4‐gland exploration is the preferred operative strategy in cases with discordant or negative preoperative localization studies.^1^, ^2^

## CONFLICT OF INTEREST

None to declare.

## AUTHOR CONTRIBUTIONS

AK: designed and implemented the study, or wrote the manuscript. DL: designed or critically reviewed the manuscript.

## ETHICAL APPROVAL

There are no identification details of the patient in the studies provided, and an informed consent was taken.

## INFORMED CONSENT

Informed consent was obtained from the patient in order to use the imaging studies.

## Data Availability

All images and information regarding the case are available upon reasonable request.
